# Pneumonia Outbreak Caused by *Chlamydophila pneumoniae* among US Air Force Academy Cadets, Colorado, USA

**DOI:** 10.3201/eid2106.141394

**Published:** 2015-06

**Authors:** Kevin A. Fajardo, Shauna C. Zorich, Jameson D. Voss, Jeffrey W. Thervil

**Affiliations:** US Air Force Academy, Colorado Springs, Colorado, USA (K.A. Fajardo);; US Air Force School of Aerospace Medicine, Wright-Patterson Air Force Base, Ohio, USA (S.C. Zorich, J.D. Voss, J.W. Thervil);; Oak Ridge Institute of Science and Education, Oak Ridge, Tennessee, USA (J.W. Thervil)

**Keywords:** pneumonia, outbreak, military personnel, cadets, Chlamydophila pneumoniae, bacteria, US Air Force Academy, Colorado, United States

## Abstract

During October 2013–May 2014, there were 102 cases of pneumonia diagnosed in US Air Force Academy cadets. A total of 73% of tested nasal washes contained *Chlamydophila pneumoniae*. This agent can be considered to be present on campus settings during outbreaks with numerous, seemingly disconnected cases of relatively mild pneumonia.

*Chlamydophila pneumoniae* is the most common *Chlamydophila* species that causes human infection (*1*). It responsible for up to 20% of community-acquired pneumonia cases in elderly adults (*1*). In recent years, *C. pneumoniae* has also been identified in outbreaks of pneumonia among younger age groups in a variety of close-quarters living environments, including military installations, prisons, universities, and single-family households (*2**–**6*).

We report the findings of our investigation into an outbreak of 102 cases of pneumonia at the US Air Force Academy, Colorado Springs, CO, USA. Laboratory testing identified *C. pneumoniae* as the likely causative pathogen.

## The Study

The US Air Force Academy houses ≈4,000 cadets. The cadet population is composed of approximately equal-sized freshman, sophomore, junior, and senior classes. Members of each class year are randomly distributed to 1 of 40 cadet squadrons (numbered 1–40). Each squadron is composed of ≈100 cadets of both sexes. Approximately 80% of cadets are men. Ten squadrons are grouped together to form 1 of 4 cadet groups. All cadets receive their health care at the cadet clinic or other military installations.

In October 2013, a cluster of radiographic-confirmed cases of pneumonia was identified as part of routine medical surveillance by the preventive medicine staff at the US Air Force Academy. Nine cases of pneumonia were diagnosed in football team members in that month. In comparison, only 8 cases of pneumonia were diagnosed in the entire cadet population during the previous academic year. Although the incidence of mild upper respiratory infections was relatively high at the time of this cluster, cases of pneumonia other than in football players were not identified.

Laboratory testing ruled out *Streptococcus pyogenes*, influenza virus, and *Legionella pneumophila* as possible infectious etiologies in these cases. The following case definition was used to identify additional cases: any cadet receiving diagnostic codes from the International Classification of Diseases, 9th Revision, for bacterial pneumonia (482, 483, 484, 485, or 486) and who had radiographic confirmation of an acute pulmonary process.

In November 2013, the first case of pneumonia outside the football team was diagnosed. The primary care providers of the cadet medical clinic were encouraged to collect nasal wash samples from any cadets with upper respiratory infection symptoms, including those with pneumonia, for further laboratory testing. All samples were sent to the US Air Force School of Aerospace Medicine Epidemiology Laboratory (Wright-Patterson Air Force Base, OH, USA), which is a Department of Defense center for febrile respiratory illness surveillance.

Infection control guidance was developed by US Air Force Academy preventive medicine staff and disseminated to all cadets. Because all cadets reside in dormitory buildings, a close-quarters living environment, the recommended preventive measures focused on reinforcement of personal hygienic practices, social distancing, common contact surface decontamination, and wearing of surgical masks.

**Figure 1:**
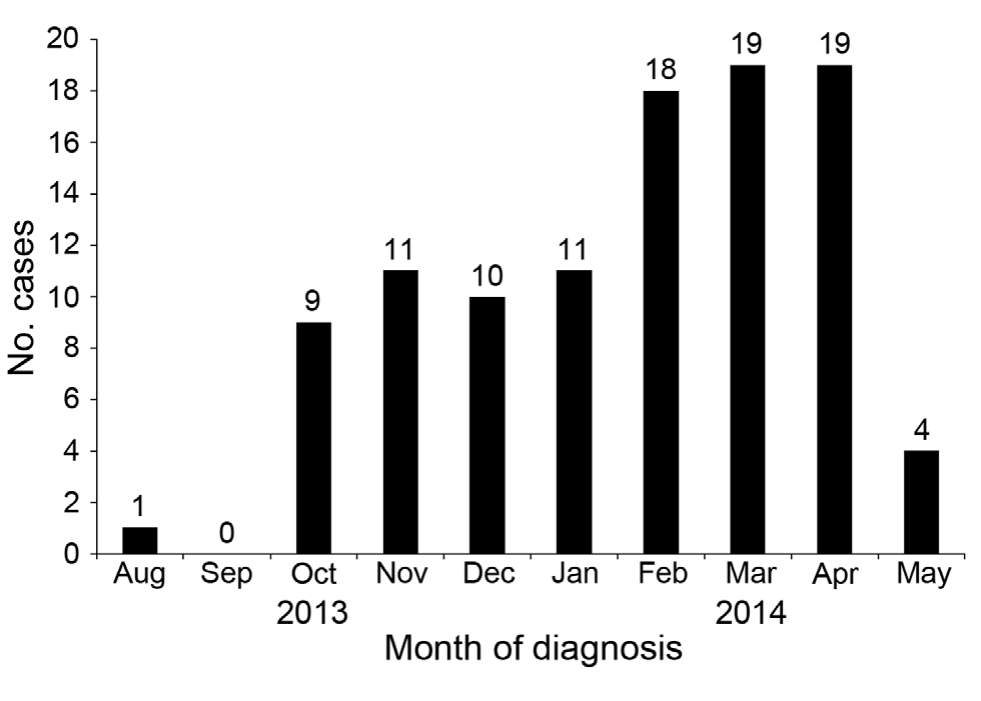
Chest radiograph–confirmed cases of pneumonia in cadets, US Air Force Academy, Colorado, USA, August 2013–May 2014.

The outbreak lasted through May 2014, and a total of 102 cases of pneumonia were identified in US Air Force Academy cadets (Figure 1). Pneumonia was diagnosed in 74 male (73%) and 28 female (27%) cadets; cases were identified in members of every class year and cadet group (Table 1). Although no major differences in attack rate were noted among cadet groups, freshmen and juniors had higher attack rates than sophomores and seniors (Table 1). For freshmen, this finding is consistent with traditional military training risk factors because freshman undergo the most physically demanding training. It is unclear why the junior class had a similar incidence of pneumonia, although this finding might reflect a higher level of social contact outside the dormitory environment for this group.

**Table 1:** Pneumonia case distribution and attack rates for cadets, US Air Force Academy, Colorado, USA, August 2013–May 2014*

Group	Men, no. (%)	Women, no. (%)	No. cases	Total population	Attack rate, %	p value by χ^2^ test of homogeneity
Class, y						
Senior, 2014	12 (71)	5 (29)	17	1,131	1.5	0.01†
Junior, 2015	23 (77)	7 (23)	30	884	3.4	
Sophomore, 2016	14 (70)	6 (30)	20	886	2.3	
Freshman, 2017	25 (71)	10 (29)	35	1,025	3.4	
Cadet group						
1	17 (77)	5 (23)	22	972	2.3	0.45‡
2	18 (78)	5 (22)	23	962	2.4	
3	26 (79)	7 (21)	33	1,002	3.3	
4	13 (54)	11 (46)	24	990	2.4	

*Group 1, squadron 1–10; group 2, squadron 11–20; group 3, squadron 21–30; group 4, squadron 31–40. †Probability of random distribution among class years. ‡Probability of random distribution among cadet groups.

Clinically, all cadets with pneumonia reported generalized, mild upper respiratory tract infection (URI) symptoms, such as cough, sore throat, and headache at the time of initial presentation. Chest radiographs showed acute, unilateral lobar consolidation for all patients. Only 4 cadets (5%) had documented evidence of fever at the time of presentation. Most cadets with pneumonia (101/102) required only a standard course of oral azithromycin therapy. One cadet, who had a pulmonary abscess, required hospitalization. He was given intravenous antimicrobial drugs and discharged from the hospital after 4 days.

Sixty-eight nasal wash samples from cadets were tested at US Air Force School of Aerospace Medicine Epidemiology Laboratory. Each specimen was tested by viral culture and real-time reverse transcription PCR for influenza virus. Influenza virus–negative specimens was then tested by using multiplex PCR testing; this PCR can identify many common respiratory pathogens, including *C. pneumoniae* (Table 2). Of the 68 samples, 15 were from cadets with pneumonia (Table 2). Of these 15 samples, 11 (73%) were positive for *C. pneumoniae*, 1 (7%) was positive for influenza A (H1N1) virus, and 3 (20%) were negative for both pathogens (Table 2).

**Table 2:** Laboratory results for 68 cadet nasal wash specimens, US Air Force Academy, Colorado, USA, August 2013–May 2014

Bacteria or virus	No. (%) positive	
	Cadets with pneumonia, n = 15	Cadets without pneumonia, n = 53
*Chlamydophila pneumonia*	11 (73)	19 (36)
Influenza A(H1N1) virus	1 (7)	8 (15)
Adenovirus	0	2 (4)
Rhinovirus/enterovirus	0	1 (2)
Coronavirus	0	1 (2)
Human metapneumovirus	0	1 (2)
Parainfluenza viruses 1–4	0	0
Respiratory syncytial virus	0	0
*Bordetella pertussis*	0	0
*Mycoplasma pneumoniae*	0	0

Fifty-three nasal wash specimens were collected from cadets who had URI symptoms but were not given a diagnosis of pneumonia. Of these 53 specimens, 19 (36%) were positive for *C. pneumoniae* (Table 2). None of the 53 cadets from whom these samples were obtained received antimicrobial drug therapy, and all recovered without complications.

**Figure 2:**
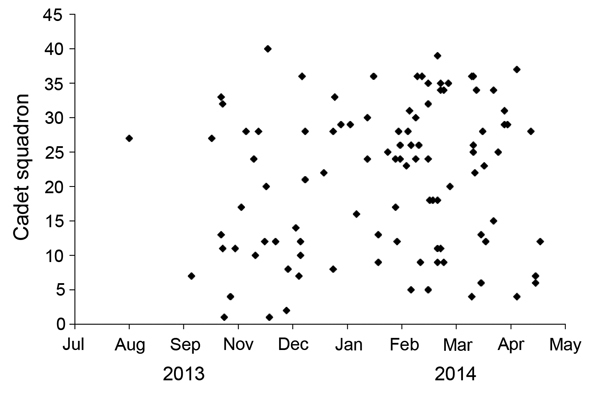
Scatter plot of pneumonia cases (diamonds) in cadets, by squadron, US Air Force Academy, Colorado, USA, August 2013–May 2014.

When charted on the basis of cadet squadron assigned, incident pneumonia cases were generally scattered throughout the cadet population (Figure 2). Only brief, self-limited clustering of cases was noted. For example, in February 2014, three squadrons had multiple cases diagnosed within a few weeks of each other (squadrons 24, 26, and 35). However, incident cases abruptly ended in these squadrons, which made it difficult to justify a large-scale antimicrobial drug prophylaxis campaign. Antimicrobial drug prophylaxis of close contacts of cadets with pneumonia was also of unclear benefit because we observed no evidence of roommate-to-roommate transmission.

The last case was diagnosed on May 15, 2014, although the surveillance period extended through July 31, 2014. Although aggressive reinforcement of infection control measures continued throughout the outbreak, the abrupt cessation of incident cases was more likely caused by the efflux of cadets off base after senior graduation and the start of underclassman summer activities.

## Conclusions

Recent evidence supports *C. pneumoniae* as an increasingly common cause of outbreaks of community-acquired pneumonia, particularly in close-quarters living environments (*2**–**6*). The outbreak described supports this finding, and laboratory studies confirmed the presence of *C. pneumoniae* in 11 (73%) of 15 nasal wash samples from cadets given a diagnosis of pneumonia. Furthermore, 19 (36%) of 53 cadets with acute URI symptoms, but who were not given a diagnosis of pneumonia, were also positive for *C. pneumoniae*. This finding might indicate that mild URI symptoms, rather than frank pneumonia, predominate in *C. pneumoniae* outbreaks. The long incubation period for infection with *C. pneumoniae*, estimated to be <4 weeks (*7*), also probably contributed to the difficulty in containing the outbreak because case diagnoses were generally made in a random pattern. Thus, the preventive intervention relied primarily on reinforcement of basic personal hygienic practices.

We suspect that routine testing for *C. pneumoniae* in outbreak situations is rare. Therefore, it is possible that *C. pneumoniae* commonly emerges on college campuses, in prisons, and other military training environments without any reporting. There are several reasons to be concerned about emergence of *C. pneumoniae* in these settings. First, unique transmission characteristics (incubation period, asymptomatic carriage) can lead to diagnostic uncertainty, which enables outbreaks to be sustained for long durations without a clear method for control. Second, if *C. pneumoniae* is not considered within the differential diagnosis, unnecessary testing for other pathogens might be conducted. Third, even if the acute illness is mild, *C. pneumoniae* has been linked with numerous chronic diseases (e.g., atherosclerosis [*8*] and asthma [*9*]).
